# Diffusion Magnetic Resonance Imaging and Human Reward System Research: A Bibliometric Analysis and Visualisation of Current Research Trends

**DOI:** 10.21315/mjms2024.31.4.9

**Published:** 2024-08-27

**Authors:** Asma Hayati Ahmad, Siti Hajar Zabri, Siti Mariam Roslan, Nur Ayunie Ayob, Aini Ismafairus Abd Hamid, Nur Hartini Mohd Taib, Nasibah Mohamad, Zahiruddin Othman, Sofina Tamam, Aleya Aziz Marzuki, Rahimah Zakaria

**Affiliations:** 1 Department of Physiology, School of Medical Sciences, Universiti Sains Malaysia, Kelantan, Malaysia; 2 Department of Neuroscience, School of Medical Sciences, Universiti Sains Malaysia, Kelantan, Malaysia; 3 Department of Radiology, School of Medical Sciences, Universiti Sains Malaysia, Kelantan, Malaysia; 4 Department of Psychiatry, School of Medical Sciences, Universiti Sains Malaysia, Kelantan, Malaysia; 5 Faculty of Science and Technology, Universiti Sains Islam Malaysia, Nilai, Malaysia; 6 School of Medical and Life Sciences, Sunway University, Petaling Jaya, Malaysia; 7 Brain & Behaviour Cluster, School of Medical Sciences, Universiti Sains Malaysia, Kelantan, Malaysia

**Keywords:** diffusion magnetic resonance imaging, humans, brain, reward, bibliometrics

## Abstract

**Background:**

The human reward system has been extensively studied using neuroimaging. This bibliometric analysis aimed to determine the global trend in diffusion magnetic resonance imaging (dMRI) and human reward research in terms of the number of documents, the most active countries and their collaborating countries, the top journals and institutions, the most prominent authors and most cited articles, and research hotspots.

**Methods:**

The research datasets were acquired from the Scopus database. The search terms used were ‘reward’ AND ‘human’ AND ‘diffusion imaging’ OR ‘diffusion tensor imaging’ OR ‘diffusion MRI’ OR ‘diffusion-weighted imaging’ OR ‘tractography’ in the abstract, article title and keywords. A total of 336 publications were analysed using Harzing’s Publish or Perish and VOSviewer software.

**Results:**

The results revealed an upward trend in the number of publications with the highest number of articles in 2020 and 2022. Most publications were limited to countries, authors, and institutions in the USA, China and Europe. Bracht, Coenen, Wiest, Federspiel and Feng were among the top authors from Switzerland, Germany and the UK. *Neuroimage*, *Neuroimage Clinical, Frontiers in Human Neuroscience, Human Brain Mapping*, and the *Journal of Neuroscience* were the top journals. Among the top articles, six were reviews and four were original articles, while the top keywords in human reward research were ‘diffusion MRI’, ‘adolescence’, ‘depression’ and ‘reward-related brain areas’.

**Conclusion:**

These findings may serve as researchers’ references to find collaborative authors, relevant journals, cooperative countries/institutions, and hot topics related to dMRI and reward research.

## Introduction

The reward system is a group of brain structures and neural pathways that are involved in reward-related cognition, such as associative learning (primarily classical conditioning and operant reinforcement), incentive salience (i.e. motivation and ‘wanting’, desire for or craving a reward) and positively valenced emotions, particularly pleasure-related emotions (i.e. hedonic ‘liking’) (1, 2). Neuroimaging techniques, such as functional magnetic resonance imaging (fMRI) and diffusion magnetic resonance imaging (dMRI), have been widely used to study the human reward system (3–5).

dMRI maps white matter pathways in the brain in vivo by measuring the diffusivity of water molecules in brain tissues. Diffusion tensor imaging (DTI), originally proposed by Basser et al. (6, 7), maps and characterises the three-dimensional diffusion of water in imaged tissues. It does not require contrast dyes, the scan time is short and it can be performed on almost all modern MR scanners (8).

The first medical literature reporting the use of dMRI on brain abnormalities was a study on patients who had suffered mild brain trauma. The researchers compared the lesioned brain to normal control subjects or to the uninvolved side of the injured patients’ brain (9). Besides its application in traumatic brain injury (10), dMRI has been used to aid in the diagnosis, prognosis and classification of brain disorders, such as stroke, brain tumours, neurodegenerative diseases, developmental disorders, movement disorders and neuropsychiatric disorders (11). It is also used to assess various neurological conditions, such as acute ischaemic stroke (12), multiple sclerosis (13, 14), schizophrenia (15), autism (16) and ageing (17). In anatomical research, it has been used to examine the structure of the language network (18, 19), the asymmetry of white matter in twins and siblings (20), and the location, asymmetry, and variability of fibre tracts (21). It has also been applied in neurosurgical planning, navigation (22–25) and predicting postoperative outcome (26). Both dMRI and fMRI have been used to model the human ‘connectome’ by analysing structural and functional brain connectivity (27, 28).

There have been several bibliometric analyses of neuroimaging. The first article found was ‘The 100 most cited articles in neuroimaging: A bibliometric analysis’ (29). Similar articles included ‘A bibliometric review of research trends in neuroimaging’ (30), ‘The most mentioned neuroimaging articles in online media: A bibliometric analysis of the top 100 articles with the highest Altimetric Attention Scores’ (31) and ‘Most common publication types of neuroimaging literature: Papers with high levels of evidence are on the rise’ (32). Bibliometric analyses of the application of neuroimaging in specific diseases, such as psychiatric disorders (33), cerebral palsy (34) and white matter hyperintensities (35), have also been published.

The aim of this bibliometric analysis was to determine the global trend in dMRI and human reward research in terms of the number of documents, the most active countries and their collaborating countries, the top journals and institutions, the most prominent authors and most cited articles, and research hotspots using a bibliometric approach.

## Methods

**Figure 1:**
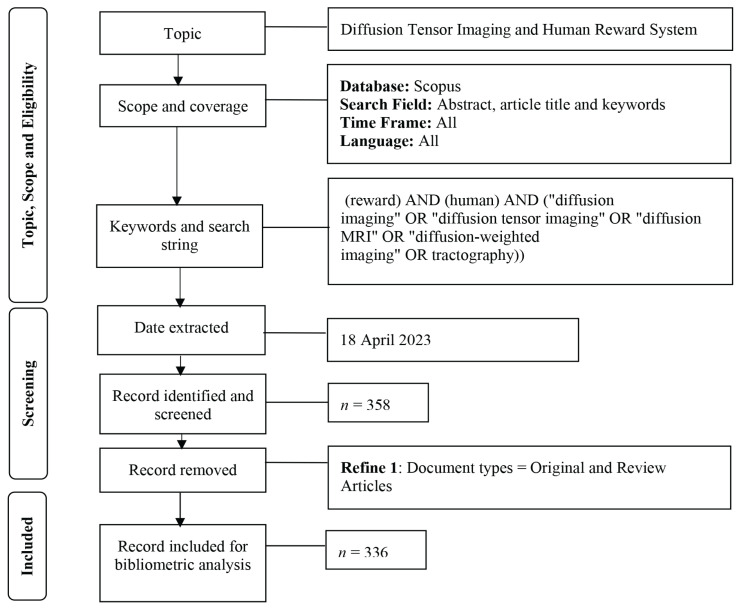
Search strategy used in this study (38)

Data were retrieved from the Scopus database on 18 April 2023. Scopus was employed in this study because it houses the world’s largest and most comprehensive collection of scientific resources (36, 37). Our search was based on PRISMA guidelines (38), as indicated in Figure 1.

The search terms ‘reward’ AND ‘human’ AND ‘diffusion imaging’ OR ‘diffusion tensor imaging’ OR ‘diffusion MRI’ OR ‘diffusion-weighted imaging’ OR ‘tractography’ in the abstract, article title and keywords were used to search for relevant articles. A total of 358 articles were identified based on the search terms. After further exclusion of certain document types and limiting the scope to only articles and review articles, 336 articles were finally analysed.

The retrieved data were used to plot a graph of publication growth over time, and citation metrics of scientific articles published related to dMRI and human reward were calculated using Harzing’s Publish or Perish software. A thesaurus file was created to incorporate words and clean the data for author keyword analysis, for example, to combine keywords such as diffusion tensor imaging, diffusion-weighted imaging, and diffusion imaging into a common term ‘diffusion MRI’. From the thesaurus file, every meaningful text mining result was saved as a map file using VOSviewer software (version 1.6.15). This software was used to visualise bibliometric networks presented as a network visualisation map. In this network map, the colour, circle size and thickness of connecting lines represent units belonging to one cluster or group, size of productivity or citations and relative link (collaboration) strength, respectively (39). In this study, we performed country co-authorship and co-occurrence author keyword analyses to create maps showing the networks.

## Results

### Publication Trends in dMRI and Human Reward Research

**Figure 2:**
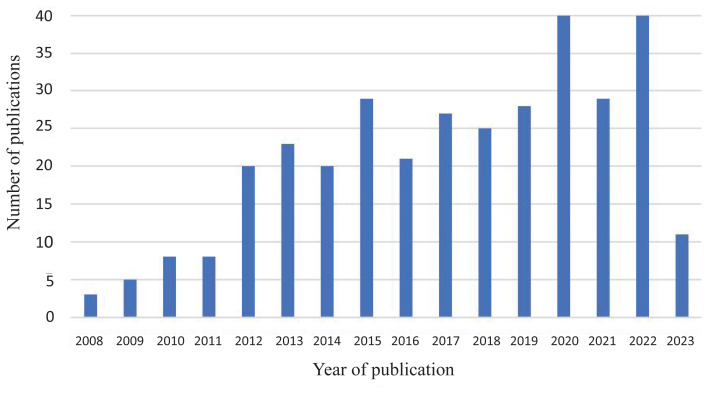
The trend of publications in dMRI and human reward research

Figure 2 shows the annual trends in publications. Three articles were published in 2008, and increasing growth was found in the following years, with slight dips in 2014, 2016, 2018 and 2021. The years 2020 and 2022 recorded the highest number of publications (40).

### Countries Active in dMRI and Human Reward Research

A total of 40 countries have contributed to the area of dMRI and human reward research. The USA published the largest number of articles (*n* = 186), followed by the UK with 51 articles, Germany with 46, China with 36, Italy with 20, Switzerland with 18, Canada and the Netherlands with 17 articles each, France with 14 and Spain with 12 articles. The USA had the highest H-index (*h* = 50), followed by the UK (*h* = 27) and Germany (*h* = 26). Although China and Italy published more articles, they had fewer total citations than Switzerland, Canada and the Netherlands. Switzerland also had a higher H-index than the other four countries (Table 1).

**Figure 3:**
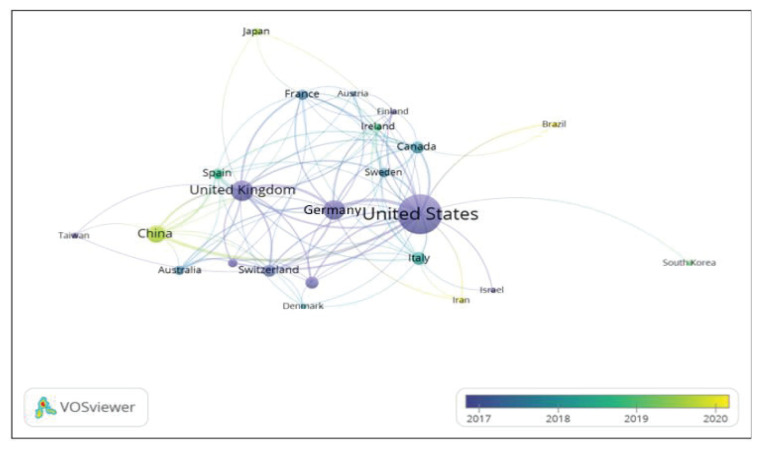
The country co-authorship network of publication with a minimum number of three documents to a country

Examining country co-authorship is a key way to represent the extent of communication between countries in this research area. In the VOSviewer network visualisation map, the connections between nodes represent cooperative relationships between countries. The distance between the nodes and the thickness of the connection represents the strength of the relationship and the co-occurrence between the countries, respectively. Figure 3 shows the countries’ co-authorship networks of publications. In the earlier phase, close collaborations are seen between the USA, as the main country that contributes to dMRI and human reward research, and the UK and other European countries. Towards the latter phase (2019 onwards), other countries besides the USA and Europe, such as China, Japan and Brazil, can be seen contributing to the research area and entering into collaborations.

### Main Journals Publishing dMRI and Human Reward Research

All studies were published in one or more of 140 journals. The top 10 journals are shown in Table 2. The journal with the highest number of publications was *Neuroimage* with a total of 15 articles (10.71%), followed by *Neuroimage Clinical* with 14 articles (10%). *Frontiers in Human Neuroscience, Human Brain Mapping, Journal of Neuroscience* and *PLoS ONE* each published 11 (7.86%) articles. These six journals account for more than half of all articles published on dMRI in human reward research.

### Main Affiliations in dMRI and Human Reward Research

The 10 most productive institutions in the field of dMRI and human reward are summarised in Table 3. All the institutions are in the USA, China and the UK. Harvard Medical School published the largest number of articles (14.6%), followed by the University of California Los Angeles (12.4%). The Ministry of Education of China, Oxford University’s Medical Sciences Division, University College London and Massachusetts General Hospital each published 10.2% of the articles.

### Analysis of Main Author Contributions

The 10 most productive authors in the dMRI and human reward area are shown in Table 4. Bracht and Coenen published the highest number of articles (nine articles each), followed by Wiest (seven articles). Federspiel, Feng, Frank, Huang, Olson, Schlaepfer and Walther published six articles each. Four of the top 10 authors were from Switzerland, while the rest were from Germany, the USA, the UK and China.

Table 5 shows the 10 most cited articles in terms of authors, titles, years, citations (cites/year) and journal. The highest number of citations for an article in the area of dMRI and human reward research was 1,369. *Brain* published two of the 10 most cited articles, while *Annals of the New York Academy of Sciences, Journal of Neuroscience, Frontiers in Human Neuroscience, American Journal of Psychiatry, Journal of Neuropsychiatry and Clinical Neurosciences, Brain Sciences, Proceedings of the National Academy of Sciences* and E*uropean Neuropsychopharmacology* each published one article that made it to the top 10 most cited articles in this field. Among the 10 articles, six were Review Articles and four were Original Articles. All the articles were published between 2008 and 2015, with 3 of 10 published in 2012. All 10 articles were co-authored and the average number of authors was 4.5.

### Keyword Analysis of Research Hotspots

Keyword co-occurrence effectively reflects research hotspots in a research field. Table 6 shows the 13 most commonly used keywords in the field of dMRI and human reward research. ‘Diffusion MRI’ is the most recurring keyword (106 occurrences), followed by ‘reward’ (45 occurrences), ‘white matter’ (37 occurrences), ‘neuroimaging’ (28 occurrences), ‘magnetic resonance imaging’ (27 occurrences) and ‘functional magnetic resonance imaging’ (26 occurrences). The term ‘diffusion MRI’ comprises all its related terms as compiled in the thesaurus function in VOSviewer software and therefore includes ‘diffusion tensor imaging’, ‘DTI’ and ‘diffusion-weighted imaging’. In addition, the table displays total link strength, which indicates the importance of a keyword in the research area, as a higher value indicates more linkages with others (39).

**Figure 4:**
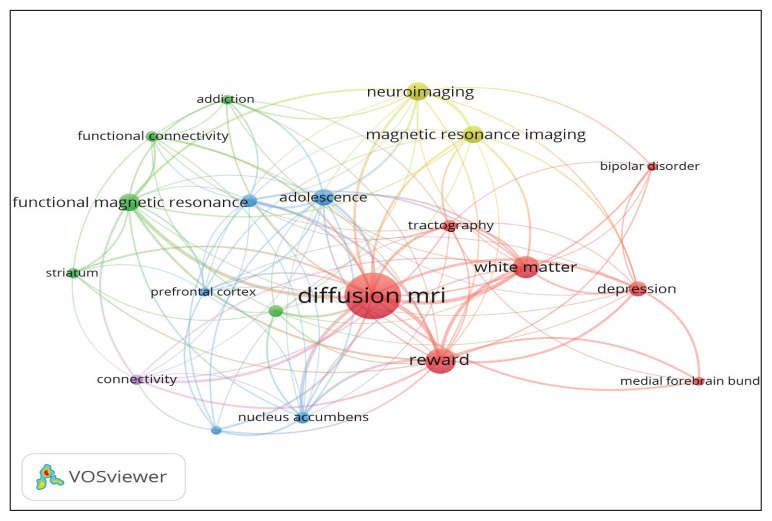
The author keywords co-occurrence network for dMRI and human reward research

In Figure 4, which depicts research hotspots, the larger nodes and fonts generally reflect greater weight of the keyword. A shorter distance and thicker line, respectively, reflect a stronger relationship between two nodes and more frequent co-occurrence of two keywords.

## Discussion

Bibliometric analysis is a tool for research evaluation (40, 41). Results from bibliometric analyses are frequently used to support decisions on research policies, research grants, job opportunities and promotions as well as to guide and support research projects based on the most relevant scientific literature (40, 42). To the best of our knowledge, this study is the first to report current research trends in dMRI and the human reward system based on bibliometric analysis and visualisation.

In this study, the results showed an upward trend with a stable rise in the number of publications, indicating increasing efforts and explorations made using dMRI in the human reward system. The USA has published the highest number of articles, followed by the United Kingdom, Germany, China, Italy, Switzerland, Canada, the Netherlands, France and Spain. Yeung et al. (43) reported similar findings of significant growth in neuroimaging literature from 2003 to 2014, with North America and Europe being the main contributors.

The top productive institutions in the field of dMRI and human reward research were Harvard Medical School, University of California Los Angeles, the Ministry of Education of China, the University of Oxford’s Medical Sciences Division and University College London. These institutions are in the USA, China and the United Kingdom, consistent with the results of the top most productive countries. Similar findings were noted in bibliometric analyses of neuroimaging (30), in which Harvard University was the leading institution in the USA and University College London in the UK. These findings indicate that worldwide research in dMRI and the human reward system was concentrated mainly in North America and Europe, while China has been emerging in many research areas, such as neuroscience (43) and neuroimaging (30, 31).

The H-index, which measures both the productivity and citation impact of publications, is a reliable and authentic parameter for academic evaluation. Based on country, the USA ranked first, with the highest H-index. The UK ranked second, followed by Germany, China, Italy, Switzerland, Canada, the Netherlands, France and Spain. Switzerland was among the top 10 countries and authors from that country were among the most productive authors apart from Germany and the USA. China, despite being relatively new in the publication of articles in this area, has emerged among the top five countries with regard to H-index.

This study reveals *Neuroimage* (*Neuroimage Clinical under Neuroimage*) as the most popular journal, followed by *Frontiers in Human Neuroscience*, *Human Brain Mapping* and *The Journal of Neuroscience*. However, in terms of total citations, *The Journal of Neuroscience* and *Brain* ranked first and second, respectively. One possible reason could be that the most cited articles were published by these journals. In addition, the articles were published between 2008 and 2014, thus allowing adequate time to be cited.

In terms of keyword analysis, ‘diffusion MRI’ was the most recurring keyword, followed by ‘reward’, ‘white matter’, ‘neuroimaging’, ‘magnetic resonance imaging’ and ‘functional magnetic resonance imaging’. The recurrence of the keyword ‘adolescence’ signifies the developmental phase characterised by reward-seeking and risk-taking behaviours with accompanying changes in the reward circuitry (44, 45). Other top keywords, such as ‘nucleus accumbens’ and ‘orbitofrontal cortex’, suggest the importance of these areas in the human reward system.

The orbitofrontal cortex (OFC) plays a critical role in processing salience and magnitude of rewards (46–48) and in integrating reward information based on its strong anatomical connection with reward-related regions, namely sensory, limbic and ventral striatal cortex (49, 50). Another core region in reward-related processing is the nucleus accumbens (NAcc), which is part of the ventral striatum (51–53). It is an essential element of the brain’s reward circuit (54) and is largely responsible for mediating hedonic perception of rewards. In addition to the perception of rewards, NAcc takes on a role as a modulator in motivation-related behaviour, which may influence several symptoms of depression, such as lack of motivation, anergia or psychomotor slowing (55), thus explaining the appearance of ‘depression’ among the top keywords.

## Conclusion

The bibliometric analysis of dMRI and human reward research showed an upward trend in the number of publications, with most articles coming from the USA, while China is an emerging country in this field. The top keywords were those related to dMRI and reward-related brain areas, as well as ‘adolescence’ and ‘depression’. These results may serve as a reference for researchers seeking collaborative authors, relevant journals, cooperative countries/institutions and hot topics related to dMRI and human reward research.

## Figures and Tables

**Table 1:** Top 10 countries contributing to the publications in human reward research

Country	TP	NCP	TC	C/P	C/CP	*h*	*g*
USA	186	186	9,481	50.97	50.97	50	94
UK	51	50	3,697	72.49	73.94	27	51
Germany	46	45	2,695	58.59	59.89	26	45
China	36	32	656	18.22	20.50	13	25
Italy	20	19	405	20.25	21.32	11	19
Switzerland	18	18	896	49.78	49.78	14	18
Canada	17	13	721	42.41	55.46	9	17
Netherlands	17	16	875	51.47	54.69	10	16
France	14	14	628	44.86	44.86	9	14
Spain	12	12	303	25.25	25.25	10	12

Note: TP = total number of publications; NCP = number of cited publications; TC = total citations; C/P = average citations per publication; C/CP = average citations per cited publication; *h* = H-index; *g* = g-index

**Table 2:** Top 10 journals in dMRI and human reward research

Source title	TP	TC	Publisher	Cite score	SJR 2021	SNIP 2021
*Neuroimage*	15	458	Elsevier	11.2	2.746	2.099
*Neuroimage Clinical*	14	317	Elsevier	8.2	1.418	1.454
*Frontiers in Human Neuroscience*	11	721	Frontiers Media S.A.	4.6	0.859	1.249
*Human Brain Mapping*	11	297	Wiley-Blackwell	8.3	1.719	1.551
*Journal of Neuroscience*	11	1,357	Society of Neuroscience	10.2	2.691	1.721
*PLoS ONE*	11	456	Public Library of Science	5.6	0.852	1.368
*Cerebral Cortex*	9	297	Oxford University Press	8.7	2.07	1.436
*Brain*	9	1,180	Oxford University Press	19.7	4.573	3.403
*Brain Imaging and Behavior*	7	202	Springer Nature	5.9	0.991	1.057
*Developmental Cognitive Neuroscience*	7	187	Elsevier	10.2	2.352	1.940

Note: TP = total number of publications; TC = total citations; SJR = Scientific Journal Ranking; SNIP = Source Normalised Impact per Paper

**Table 3:** Top 10 productive institutions in dMRI and human reward research

Rank	Affiliation	Country	TP
1	Harvard Medical School	USA	20
2	University of California, Los Angeles	USA	17
3	Ministry of Education, China	China	14
4	University of Oxford Medical Sciences Division	UK	14
5	University College London	UK	14
6	Massachusetts General Hospital	USA	14
7	Stanford University	USA	12
8	University of Southern California	USA	12
9	University of Oxford	UK	10
10	University of California, San Diego	USA	10

Note: TP = total number of publications

**Table 4:** Top 10 productive authors in dMRI and human reward research

Rank	Authors	TP	Affiliation
1	Bracht	9	UniversitätsSpital Bern, Bern, Switzerland
2	Coenen	9	Universitäts Klinikum Freiburg und Medizinische Fakultät, Freiburg im Breisgau, Germany
3	Wiest	7	University of Bern, Bern, Switzerland
4	Federspiel	6	UniversitätsSpital Bern, Bern, Switzerland
5	Feng	6	Department of Computer Science, University of Warwick, Coventry, United Kingdom
6	Frank	6	University of California, San Diego, San Diego, United States
7	Huang	6	Shanghai Key Laboratory of Brain Functional Genomics (Ministry of Education), School of Psychology and Cognitive Science, East China Normal University, Shanghai, China
8	Olson	6	Temple University, Philadelphia, United States
9	Schlaepfer	6	Universitäts Klinikum Freiburg und Medizinische Fakultät, Freiburg im Breisgau, Germany
10	Walther	6	UniversitätsSpital Bern, Bern, Switzerland

Note: TP = total number of publications

**Table 5:** Top 10 cited articles in dMRI and human reward research

Rank	Authors	Title	Year	Cites (C/Y)	Journal
1	Casey, Jones, Hare	The adolescent brain	2008	1,369 (91.27)	*Annals of the New York Academy of Sciences*
2	Beckmann, Johansen-Berg, Rushworth	Connectivity-based parcellation of human cingulate cortex and its relation to functional specialisation	2009	601 (42.93)	*Journal of Neuroscience*
3	Von Der Heide, Skipper, Klobusicky, Olson	Dissecting the uncinate fasciculus: disorders, controversies and a hypothesis	2013	498 (49.8)	*Brain*
4	Hart, Rubia	Neuroimaging of child abuse: A critical review	2012	439 (39.91)	*Frontiers in Human Neuroscience*
5	Phillips, Swartz	A critical appraisal of neuroimaging studies of bipolar disorder: Toward a new conceptualisation of underlying neural circuitry and a road map for future research	2014	392 (43.56)	*American Journal of Psychiatry*
6	Upadhyay, Maleki, Potter, Elman, Rudrauf, Knudsen, et al.	Alterations in brain structure and functional connectivity in prescription opioid-dependent patients	2010	270 (20.77)	*Brain*
7	Coenen, Panksepp, Hurwitz, Urbach, Mädler	Human medial forebrain bundle (MFB) and anterior thalamic radiation (ATR): Imaging of two major subcortical pathways and the dynamic balance of opposite affects in understanding depression	2012	244 (22.18)	*Journal of Neuropsychiatry and Clinical Neurosciences*
8	Kuss, Griffiths	Internet and gaming addiction: A systematic literature review of neuroimaging studies	2012	232 (21.09)	*Brain Sciences*
9	Neubert, Mars, Sallet, Rushworth	Connectivity reveals relationship of brain areas for reward-guided learning and decision making in human and monkey frontal cortex	2015	230 (28.75)	*Proceedings of the National Academy of Sciences*
10	Millan, Fone, Steckler, Horan	Negative symptoms of schizophrenia: Clinical characteristics, pathophysiological substrates, experimental models and prospects for improved treatment	2014	225 (25.0)	*European Neuropsychopharmacology*

**Table 6:** Author keywords co-occurrence (appearing 10 times or more)

Author keyword	Occurrence	Total link strength
diffusion MRI	106	136
reward	45	85
white matter	37	55
neuroimaging	28	37
magnetic resonance imaging	27	30
functional magnetic resonance imaging	26	42
adolescence	24	34
depression	21	34
impulsivity	17	29
nucleus accumbens	16	38
orbitofrontal cortex	16	19
tractography	15	24
connectivity	13	27
functional connectivity	13	13
striatum	12	12
addiction	11	15
amygdala	10	21
bipolar disorder	10	11
medial forebrain bundle	10	16
prefrontal cortex	10	18
